# Proliferation-cycle gene signatures predict immune landscape and prognosis in lung adenocarcinoma

**DOI:** 10.1007/s12672-025-04346-6

**Published:** 2025-12-31

**Authors:** Chenjing Lin, Man Zhang, Pan Sun, Yulin He, Yi Tian, Wenwen Li, Shengyan Pu, Jizhuang Luo, Kai Wang

**Affiliations:** 1 Central Laboratory, Shanghai Chest Hospital, Shanghai Jiao Tong University School of Medicine, Shanghai, People’s Republic of China; 2 Department of Chemotherapy, Fujian Provincial Geriatric Hospital, Fujian Provincial Hospital North Branch, Fuzhou, Fujian People’s Republic of China; 3 Department of Radiology, Xiangyang Hospital of Traditional Chinese Medicine, Hubei University of Chinese Medicine, Xiangyang, People’s Republic of China; 4 Precision Medicine Research Center, Clinical Research Center, Shanghai Chest Hospital, Shanghai Jiao Tong University School of Medicine, Shanghai, People’s Republic of China; 5 Department of Clinical Medical, Sichuan Provincial People’s Hospital East Sichuan Hospital & Dazhou First People’s Hospital, Dazhou, People’s Republic of China; 6 Department of Pulmonary Medicine, Shanghai Chest Hospital, Shanghai Jiao Tong University School of Medicine, Shanghai, People’s Republic of China; 7 Department of Cardiovascular Surgery, Shanghai Chest Hospital, Shanghai Jiao Tong University School of Medicine, Shanghai, People’s Republic of China; 8 Department of Thoracic Surgery, Shanghai Chest Hospital, Shanghai Jiao Tong University School of Medicine, Shanghai, People’s Republic of China

**Keywords:** LUAD, Proliferation-cycle genes, TIME, Risk stratification, Immunotherapy

## Abstract

**Background:**

Despite advancements in diagnostic techniques and therapeutic strategies, the prognosis for Lung adenocarcinoma (LUAD) patients remains poor. Cell proliferation and cycle dysregulation drive cancer via uncontrolled cell growth. These genes also modulate tumor immune microenvironment (TIME), yet the precise mechanisms in LUAD remain largely unknown.

**Methods and results:**

This study aimed to identify key proliferation-cycle genes in LUAD, characterize the TIME associated with proliferation-cycle gene signatures and assess the impact of proliferation-cycle gene signatures on immunotherapy responsiveness. We analyzed The Cancer Genome Atlas (TCGA) LUAD transcriptomic data and identified eight proliferation-cycle-related risk genes (seven up-regulated: FAP, IL2RA, ITGA2, CHORDC1, PIM2, POU3F2, CD180; one down-regulated: FKBP1B). Independent cross-validation using the National Center for Biotechnology Information (NCBI) Gene Expression Omnibus (GEO) dataset confirmed the consistent expression patterns for all eight candidate genes in LUAD tumors. A risk model based on these genes stratified patients into distinct prognostic groups, revealing: (1) Survival disparity: High-risk patients exhibited poorer overall survival (p = 6.2e−05). (2) Immunosuppressive TIME: Elevated risk scores correlated with enhanced immune infiltration (p = 2.9e−12), enriched immunosuppressive populations (Tregs), reduced cytotoxic effectors (CD8+ T cells), and up-regulated immune checkpoint molecules (PDCD1/PD-L1, CTLA4). (3) Scientific implications: Risk signatures exhibited no significant correlation with tumor mutational burden (TMB), yet uncovered novel candidate targets with therapeutic potential, meriting further mechanistic exploration.

**Conclusion:**

Proliferation-cycle gene signatures are robust biomarkers for LUAD risk stratification, prognosis, and immune landscape prediction. Their mechanistic integration into multi-dimensional oncological models could reveal previously unrecognized layers of antitumor immune regulation.

**Supplementary Information:**

The online version contains supplementary material available at 10.1007/s12672-025-04346-6.

## Introduction

The TIME is a critical determinant of immunotherapy efficacy, comprising diverse immune cells and cytokines that regulate tumor growth, progression, and therapeutic responses [1–3]. Key immune components, such as cytotoxic T cells, regulatory T cells (Tregs), and tumor-associated macrophages (TAMs)-play pivotal roles in shaping antitumor immunity. For instance, effector T cells mediate tumor cell killing upon antigen recognition [4, 5], while Tregs and M2-polarized TAMs suppress immune responses and promote metastasis [6, 7]. Immune checkpoint molecules (e.g., PD-1/PD-L1, CTLA-4) [8], as well as cell proliferation and/or cell cycle regulators (e.g., Cyclin D1) [9] further modulate TIME immunosuppression. Blocking these pathways with inhibitors (e.g., nivolumab, pembrolizumab) restores T cell activity and enhances antitumor immunity [10]. Recent studies have shed light on the intricate interplay between genes/signatures that driving cancer cell proliferation, TIME remodeling, and immune response modulation. Innovative tools like iMLGAM and PTMLS leverage multi-omics data and machine learning to predict immunotherapy outcomes, revealing key molecules such as CEP55 and B4GALT2 that modulate immune responses [11, 12]. Additionally, the Plasma cell.Sig signature, identified through pan-cancer single-cell RNA sequencing, accurately predicts immunotherapy responses, with low-risk groups exhibiting enhanced immune infiltration [3]. These findings advance personalized immunotherapy strategies and offer new therapeutic targets to improve treatment efficacy.

LUAD, the most prevalent lung cancer subtype, exhibits high mortality due to complex molecular mechanisms driving proliferation, metastasis, and immune evasion [13]. Cell proliferation and/or cell cycle-related genes, such as cyclins, cyclin-dependent kinases (CDKs), CDK inhibitors (e.g., CDKN2A/B), and TP53, are frequently dysregulated in LUAD [14–16]. Cyclin D1 overexpression drives aberrant proliferation [16], while TP53 mutations impair DNA damage surveillance, fostering genomic instability and oncogenesis [17–19]. Despite advances in understanding these genes’ roles in LUAD biology, their precise contributions to TIME modulation and therapeutic targeting remain incompletely defined.

Here, we interrogated the expression patterns of proliferation-cycle genes in LUAD and their associations with TIME characteristics, clinical outcomes, and explored their potential as immunotherapy targets. By utilizing transcriptome data of LUAD in TCGA, we identified eight prognostic risk factors and constructed a predictive model to stratify patients based on survival risk. Our findings reveal distinct TIME landscapes across risk subgroups, informing personalized immunotherapy strategies. Notably, the absence of correlation between risk patterns and TMB reveals the complexity of survival-associated factors in LUAD, highlighting the need for integrated biomarkers to guide precision oncology.

## Materials and methods

### Identification of proliferation-cycle gene signatures

RNA-seq data from 589 LUAD samples (530 tumors/59 normals) in TCGA [20] and a validation cohort (51 tumors/49 normals, GSE140343 [21]) were processed using Trimmomatic [22] for quality control, aligned to hg38 via the Spliced Transcripts Alignment to a Reference (STAR) software [23], and quantified with the RNA-Seq by Expectation Maximization (RSEM) software [24]. Protein-coding differential expressed genes (DEGs) were identified using limma [25] with thresholds of |FC|≥ 1.5 and p.adj ≤ 0.05 after log₂(TPM + 1) normalization. All p-values were adjusted via the Benjamini–Hochberg method for multiple testing correction. In DEG analysis, consensus DEGs were identified by intersecting results from the two independent cohorts (TCGA-LUAD and NCBI GSE140343) mentioned above. Key genes were experimentally validated via qPCR in multiple cell lines (H1299, H23, PC9) and computationally corroborated using NCBI GEO series GSE148036 (5 tumors/5 normals) [26] and GSE251840 (62 tumors) [27]). Given that there were relatively few normal controls, the 49 normal samples from GSE140343 were incorporated into the validation dataset. This ensures the reliability of our research results. All datasets underwent standardized processing via an integrated pipeline comprising STAR for alignment, RSEM for transcript quantification, and Transcripts Per Million (TPM) normalization for gene expression scaling, followed by ComBat harmonization to minimize batch effects. The Proliferation-Cycle Gene Signature was defined by integrating DEGs annotated to cell cycle-related GO terms (GO:0008283, cell population proliferation; GO:0007049, cell cycle), reflecting their coordinated roles in tumor growth regulation.

### Enrichment analysis

The Gene Ontology (GO) and Kyoto Encyclopedia of Genes and Genomes (KEGG) pathway enrichment of DEGs were performed using the R package clusterProfiler [28]. Gene Set Enrichment Analysis (GSEA) evaluated global pathway activity based on ranked log₂FC values [29].

### Survival analysis

Survival analysis was conducted using the R packages survival and survminer. Patient survival data, encompassing both survival duration and censoring status, were retrieved from TCGA [20]. TCGA clinical data were stratified by an extreme quantile method: gene expression thresholds were defined as the top and bottom 10% trimmed mean values (sorted by expression) to reduce the influence of outliers, with samples above/below thresholds classified as high/low expression. Risk stratification was performed using univariate Cox proportional hazards regression to estimate Hazard Ratios (HRs). Statistical significance was defined as a log-rank test p-value ≤ 0.05, serving as the threshold for identifying prognostically relevant covariates.

### Multivariate cox regression analysis

In the multivariate Cox regression analysis, all specified variables—risk.score, TNM stage, TMB, and PD-L1—were included as continuous covariates. Specifically, The risk score was derived from formula (1), TNM stage was modeled as an ordinal variable (1–4) based on clinical staging criteria, TMB was calculated using the maftools R package [30], and PD-L1 expression was quantified as log2-transformed TPM values. Multivariate Cox regression was performed using the coxph function from the survival R package to assess the prognostic impact of gene signature/biomarker while adjusting for clinical covariates. The final model was visualized with a forest plot generated by the ggforest function from the survminer R package, displaying hazard ratios (HRs) with 95% confidence intervals (CIs) for each covariate.

### Immunophenotyping and tumor immune infiltration analysis

The immune landscape was profiled using two complementary R packages: Firstly, the Estimation of STromal and Immune cells in MAlignant Tumours using Expression data (ESTIMATE) [31] was employed to quantify stromal and immune components, providing Estimate, Immune, and Stromal scores, as well as tumor purity metrics. Secondly, the Cell-type Identification By Estimating Relative Subsets Of RNA Transcripts (CIBERSORT) [32] was utilized to deconvolute the relative proportions of 22 distinct immune cell subsets within the tumor microenvironment, including CD8⁺ T cells, Tregs, macrophages (M0/M1/M2), NK cells, and myeloid dendritic cells, etc. Proportion distributions were visualized using the ggplot2 in R package. Immunophenotypes were stratified into two functional axes: (1) anti-tumor phenotype: dominated by cytotoxic effectors (activated CD8⁺ T cells, M1 macrophages, NK cells) promoting tumor clearance; and (2) pro-tumor phenotype: characterized by suppressive populations (Tregs, M2 macrophages, myeloid suppressor cells/MDSCs) secreting IL-10/TGF-β to dampen immunity. Usually, the ratio of these cells is more clinically significant and has practical prospective value, therefore it is used in this study. Key clinical metrics included: (1) CD8⁺/Treg (elevated CD8⁺/Treg ratios define immune-dominant tumors); (2) M1/M2 (Macrophage polarization emerges as a critical determinant of tumor progression, reflecting the dynamic interplay between pro-inflammatory M1 and immunosuppressive M2 phenotypes within the tumor microenvironment); (3) activated/resting NK cells (the equilibrium between activating receptor (e.g., NKG2D) and inhibitory receptor (e.g., TIGIT) dictates NK cell-mediated cytotoxicity against tumor targets/tumor-killing efficiency). These parameters were integrated to evaluate TIME-based prognostic and therapeutic potential, with implications for anti-tumor peptide/antibody therapeutics design targeting immunosuppressive axes.

### TMB stratification

TMB was calculated via maftools [30] to assess genomic instability. A binary TMB classification (high/low) was derived using mutation level thresholds: the mean value of genes ranked in the top 10% (hypermutated) and bottom 10% (hypomutated) defined mutation load cutoffs.

### Gene expression validation by qRT-PCR assay

Human lung adenocarcinoma cells (H1299, H23, PC9) and human embryonic lung fibroblast cells (MRC5) were purchased from COBIOER Biosciences Co., Ltd. (Nanjing, China). ALL experiments were carried out according to the manufacturer's protocol. Total RNA was harvested from these cells using TRIzol Reagent (Thermo Fisher Scientific, Inc.), followed by RNA purity examination and cDNA synthesis using ABscript II RT Master Mix (ABclonal, Inc.). qPCR of key genes (Table 1) was performed with PowerUP™ SYBR™ Green Master Mix (Applied Biosystems, Inc.) and primers were designed using PrimerBank [33] (Table 2; Sangon Biotech synthesis with PAGEplus method). 10 ng cDNA was used as a template. Triplicate PCR reactions were carried out in a 20 μl reaction volume following the standard cycling mode. Relative expression (ΔCt) was normalized to GAPDH, with specificity confirmed by melting curve analysis.

**Table 1:** Risk factors utilized for risk modeling

GENEID	Log_2_FC	HR	HR.ci.lower	HR.ci.upper	logrank.pvalue (univ.cox/p.adj)	logrank.pvalue (survival.fit/ p.adj)
*FAP*	1.58	1.12	1.02	1.23	0.0140/ 0.0256	0.0046/ 0.0174
*IL2RA*	1.09	1.12	1.02	1.24	0.0220/ 0.0274	0.0480/ 0.0480
*ITGA2*	1.05	1.11	1.04	1.19	0.0030/ 0.0120	0.0473/ 0.0480
*CHORDC1*	0.68	1.20	1.00	1.43	0.0480/ 0.0480	0.0476/ 0.0480
*PIM2*	0.68	1.17	1.04	1.31	0.0069/ 0.0184	0.0218/ 0.0349
*POU3F2*	0.66	1.10	1.01	1.20	0.0240/ 0.0274	0.0081/ 0.0174
*CD180*	0.59	1.20	1.06	1.35	0.0028/ 0.0120	0.0018/ 0.0144
*FKBP1B*	− 0.77	0.85	0.75	0.97	0.0160/ 0.0256	0.0087/ 0.0174

p.adj denotes the p-value adjusted using the Benjamini-Hochberg (FDR) method

**Table 2:** qRT-PCR primers used for gene expression validation of eight risk factors

Gene symbol	Forward primer	Reverse primer
*GAPDH*	ACAACTTTGGTATCGTGGAAGG	GCCATCACGCCACAGTTTC
*FAP*	CAAAGGCTGGAGCTAAGAATCC	ACTGCAAACATACTCGTTCATCA
*IL2RA*	CGCAGAATAAAAAGCGGGTCA	ACTTGTTTCGTTGTGTTCCGA
*ITGA2*	GGGAATCAGTATTACACAACGGG	CCACAACATCTATGAGGGAAGGG
*CHORDC1*	ATCTGCCTCCCTAAAACAAGC	TCCATTCTTACATGAGGTCCCA
*PIM2*	GCACTGCTATGGAAAGTGGGT	ATGGACAACTCCACGGGAATG
*POU3F2*	AAGCGGAAAAAGCGGACCT	GTGTGGTGGAGTGTCCCTAC
*CD180*	AACCTAAGCCTGAACTTCAATGG	GCCAGAGAGACTGAGTAGTAGAG
*FKBP1B*	GACGGAAGGACATTCCCCAAG	CCCATTTTGGAGCATTCCTGT

*GAPDH* is used for control

## Results

### Proliferation-cycle gene signatures in lung adenocarcinoma

**Fig. 1:**
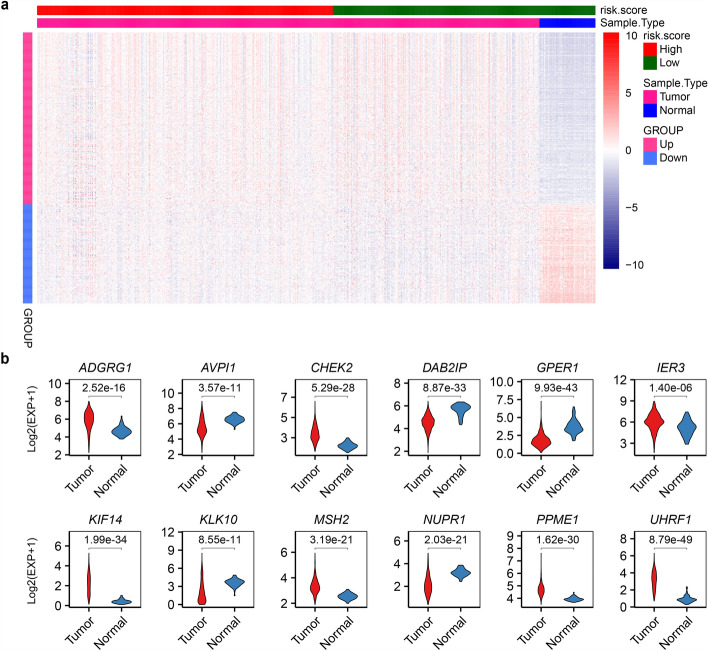
Proliferation-cycle gene dysregulation in LUAD. **a** Heatmap of 487 differentially expressed proliferation-cycle genes in LUAD vs. normal tissues. **b** Violin diagram of the 12 DEGs associated with cancer development/progression/metastasis

To elucidate the role of cell proliferation and cycle-related genes in LUAD, we analyzed their expression patterns in tumor vs. normal tissues. A total of 1859 protein-coding genes annotated with "cell population proliferation" (GO:0008283) or "cell cycle" (GO:0007049) were retrieved from UniProtKB (https://www.uniprot.org), comprising 1,263 proliferation- and 716 cycle-associated genes. Cross-validation using TCGA-LUAD and NCBI GSE140343 datasets [21] identified 487 DEGs (|FC|≥ 1.5, p.adj ≤ 0.05), including 309 up-regulated and 178 down-regulated genes (Fig. 1a, and Supplementary Table S1). Further keyword-based filtering highlighted 12 DEGs with established roles in cancer development, progression, or metastasis (UniProt annotation). These included seven up-regulated genes (*ADGRG1, CHEK2, IER3, KIF14, MSH2, PPME1,* and *UHRF1*) and five down-regulated genes (*AVPI1, DAB2IP, GPER1, KLK10,* and *NUPR1*) (Fig. 1b). Literature evidence supports their oncogenic or tumor-suppressive functions. For example, ADGRG1 promotes cancer progression and tumorigenesis [34–36]. KIF14 disrupts cell cycle regulation and drives breast cancer progression [37]. NUPR1 enhances stress-induced cancer development [38]. These gene signatures provide a functional framework for dissecting proliferation-cycle associated immune remodeling in LUAD.

### Risk model construction and validation

**Fig. 2:**
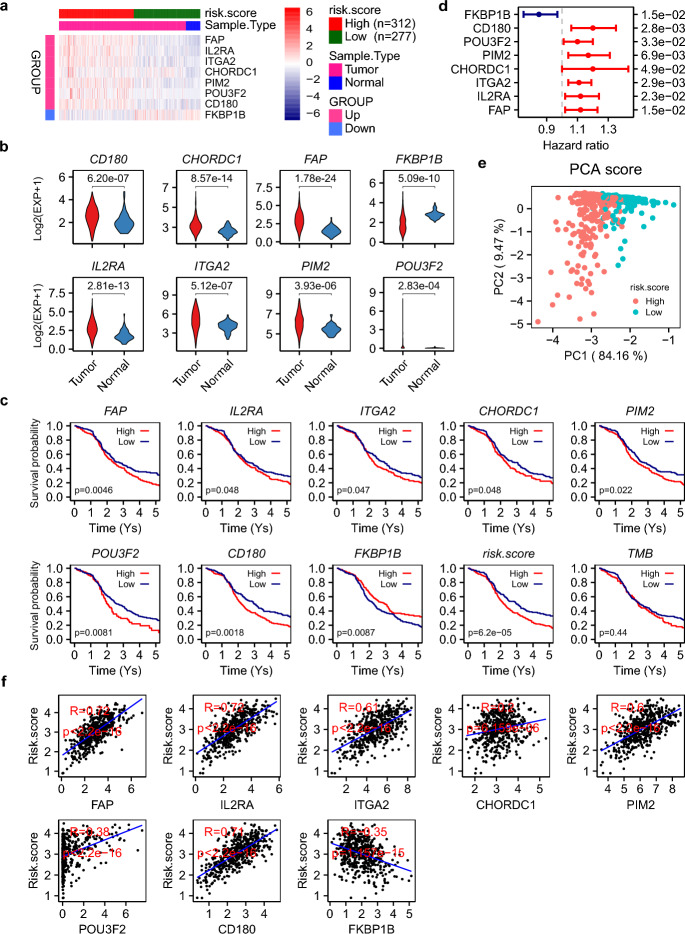
Risk model construction and prognostic validation. **a** Heatmap and **b** violin plots of eight risk factors expression. **c** Kaplan–Meier analysis stratified survival outcomes by 5-year overall survival rates. **d** Forest plot of hazard ratios from univariate cox test. **e** Dot plot depicting the high and low risk stratification of different patients, showcasing the first two components derived from PCA. **f** Correlation analysis between expressions and scores of eight risk factors

**Fig. 3:**
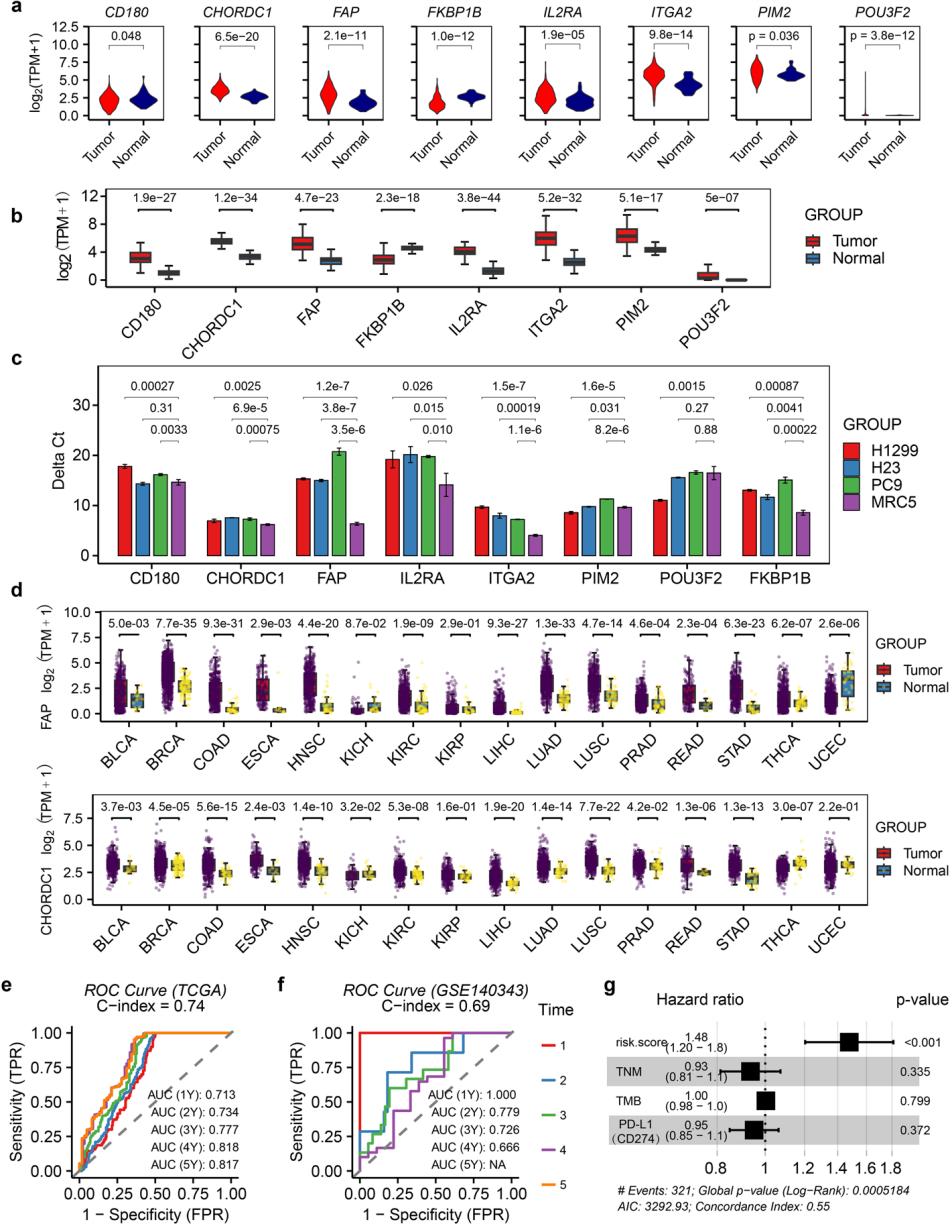
Cross-Cancer and qRT-PCR experimental validation. **a** Expression patterns of eight risk factors in LUSC. **b** Independent validation of eight risk factors using GEO datasets (GSE140343, GSE148036, GSE251840). **c** qRT-PCR validation in lung cancer cell lines (H1299, H23, PC9) vs. normal human fetal lung cells (MRC5) (n = 3 technical replicates). **d** FAP and CHORDC1 expression across 16 cancer types. **e**, **f** Prognostic performance of the risk model evaluated by ROC curves in **e** TCGA-LUAD and **f** GEO GSE140343 cohorts. **g** Prognostic comparison of the risk model with TNM stage, TMB, and PD-L1 status in LUAD using multivariate Cox regression

**Fig. 4:**
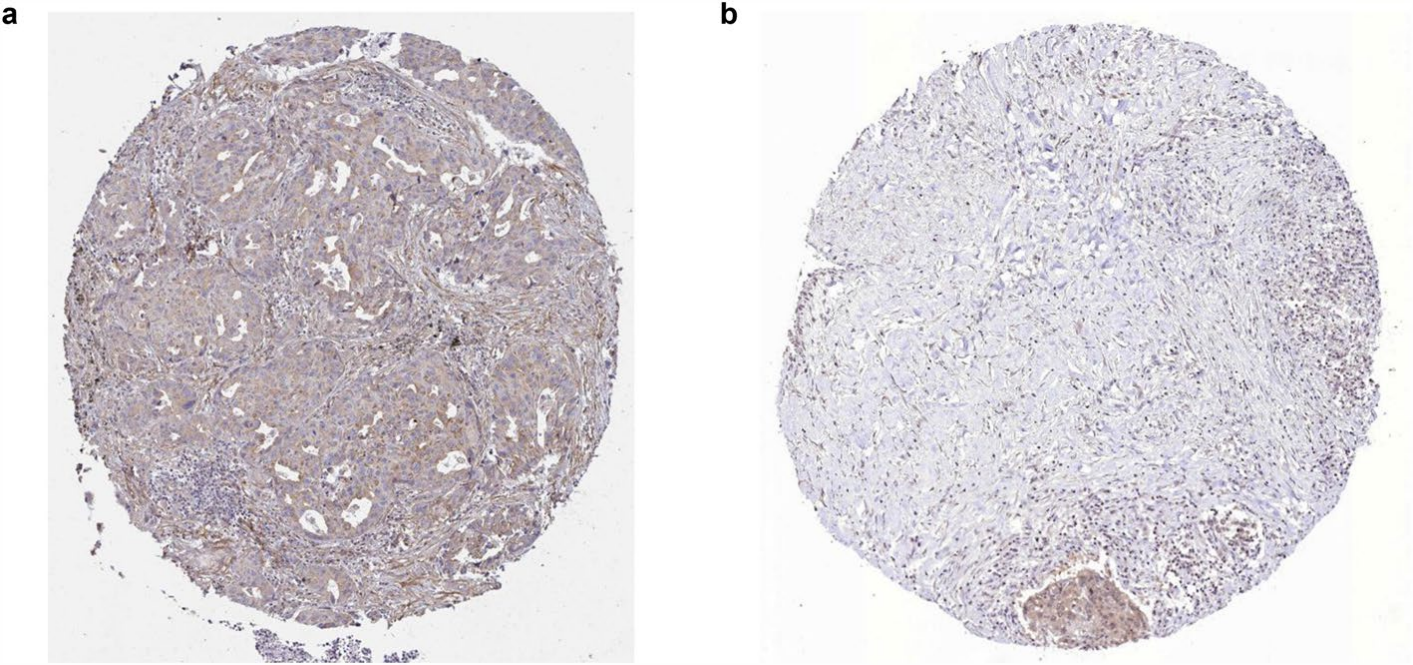
IHC evidence for FAP and CHORDC1 protein expression in LUAD tissues. **a** FAP IHC in LUAD (HPA059739). **b** CHORDC1 IHC in LUAD (HPA041040)

To assess clinical relevance, we prioritized eight proliferation-cycle genes as risk factors using survival analysis and univariate Cox regression (log-rank p ≤ 0.05, HR ≥ 1.10 for up-regulated genes, HR ≤ 0.90 for down-regulated genes) (Table 1, Fig. 2a-b). These included seven up-regulated genes (*FAP*, *IL2RA*, *ITGA2*, *CHORDC1*, *PIM2*, *POU3F2*, *CD180*) and one down-regulated gene (*FKBP1B*), highlighting that both overexpression and suppression of proliferation-cycle genes contribute to LUAD risk. Survival analysis with HR risk assessment confirmed significant prognostic differences between high- and low-risk groups (Fig. 2c-d). Cross-cancer validation using TCGA lung squamous cell carcinoma (TCGA-LUSC) cohorts demonstrated that seven out of eight risk factors (*FAP*, *IL2RA*, *ITGA2*, *CHORDC1*, *PIM2*, *POU3F2,* and *FKBP1B*) exhibited conserved expression patterns in both LUAD and LUSC (Fig. 3a). In addition, further validation using new specimens from the NCBI GEO database (GSE140343, GSE148036, and GSE251840), aligned with our expectations, affirming the significance and reliability of these genes in LUAD (Fig. 3b). qRT-PCR experiments consistently confirmed the expression patterns of *CD180, PIM2,* and *POU3F2* in at least one of the tested cell lines (H1299, H23, PC9) (Table 2, Fig. 3c). In contrast, although the remaining five genes exhibited differential expression, their regulatory directions were inconsistent with those observed in TCGA cohorts. Notably, both *FAP* and *CHORDC1* exhibited consistent dysregulation in more than half of the 16 cancer types examined (BLCA, BRCA, COAD, ESCA, HNSC, KICH, KIRC, KIRP, LIHC, LUAD, LUSC, PRAD, READ, STAD, THCA, UCEC) (Fig. 3d), suggesting their broad oncogenic roles. The Immunohistochemistry (IHC) image data from The Human Protein Atlas (https://www.proteinatlas.org) also provided solid evidence supporting the protein expression of FAP and CHORDC1 in LUAD tissues (Fig. 4a and b, HPA059739, HPA041040). None of the risk factors overlapped with previously identified tumor risk factors, thus warranting further functional studies.

A risk scoring model was developed using expression and HR data, effectively stratifying patients into high- and low-risk groups (Fig. 2a and e). A higher score indicates greater risk. Risk scores correlated positively with the seven up-regulated risk factors (*FAP*, *IL2RA*, *ITGA2*, *CHORDC1*, *PIM2*, *POU3F2*, *CD180*) and negatively with the down-regulated *FKBP1B* (Fig. 2f). All normal samples were clustered in the low-risk group (Fig. 2a), highlighting the discriminative power of the model. The risk scoring model is illustrated in formula (1):

1 $$ \left\{ {\begin{array}{*{20}c} {risk.score = \frac{{\sum _{g} E_{g} \cdot S_{g} }}{n}} \\ {S_{g} = \left\{ {\begin{array}{*{20}c} {HR,upregulatedandHR \ge 1.1} \\ { - \frac{1}{{HR}},downregulatedandHR \le 0.9} \\ \end{array} } \right.} \\ \end{array} } \right. $$

where, *E*_*g*_ is the log_2_ transformed expression value of gene g, and *S*_*g*_ is the designated symbolic variable of gene g, which is determined by HR from univariate cox analysis. *n* is the number of total genes used in risk model. For example, given the HRs from univariate cox analysis for the risk factors (FAP, IL2RA, ITGA2, CHORDC1, PIM2, POU3F2, CD180, and FKBP1B) are 1.12, 1.12, 1.11, 1.20, 1.17, 1.10, 1.20, and 0.85 respectively, the symbolic variable S_g_ can be calculated according to the described formula, yielding values of 1.12, 1.12, 1.11, 1.20, 1.17, 1.10, 1.20 (HR), and − 1.18$(-\frac{1}{HR}$) for each gene in the same order. Assuming the log_2_ transformed expression value of these genes in a LUAD patient are 3.46, 2.73, 7.31, 3.79, 5.25, 0.13, 3.07, and 0.99 respectively, then the risk score can be calculated as:

$$ {\mathrm{risk}}.{\text{score }} = {\text{ }}\left( \begin{gathered} {\mathrm{3}}.{\mathrm{46}}{\mkern 1mu} \times {\mkern 1mu} {\mathrm{1}}.{\mathrm{12}} + {\mathrm{2}}.{\mathrm{73}}{\mkern 1mu} \times {\mkern 1mu} {\mathrm{1}}.{\mathrm{12}} + {\mathrm{7}}.{\mathrm{31}}{\mkern 1mu} \times \hfill \\ {\mkern 1mu} {\mathrm{1}}.{\mathrm{11}} + {\mathrm{3}}.{\mathrm{79}}{\mkern 1mu} \times {\mkern 1mu} {\mathrm{1}}.{\mathrm{2}}0 + {\mathrm{5}}.{\mathrm{25}}{\mkern 1mu} \times {\mkern 1mu} {\mathrm{1}}.{\mathrm{17}} + \hfill \\ 0.{\mathrm{13}}{\mkern 1mu} \times {\mkern 1mu} {\mathrm{1}}.{\mathrm{1}}0 + {\mathrm{3}}.0{\mathrm{7}}{\mkern 1mu} \times {\mkern 1mu} {\mathrm{1}}.{\mathrm{2}}0 + 0.{\mathrm{99}}{\mkern 1mu} \times {\mkern 1mu} \left( { - {\mathrm{1}}.{\mathrm{18}}} \right) \hfill \\ \end{gathered} \right)/{\mathrm{8}} = {\mathrm{3}}.{\mathrm{55}} $$

The prognostic capability of the risk model was validated through two independent LUAD cohorts, specifically utilizing data from TCGA-LUAD and NCBI GEO GSE140343, both of which included survival information. For the Cox model based on TCGA-LUAD data, the Concordance Index (C-index) was calculated to be 0.74 (Fig. 3e). Furthermore, the area under the receiver operating characteristic curve (AUC ROC) for the first five years of follow-up was 0.713, 0.734, 0.777, 0.818, and 0.817, respectively. Regarding the Cox model derived from GSE140343, the C-index was 0.69 (Fig. 3f), with the AUC ROC curve for the initial 4 years of follow-up showing values of 1.000, 0.779, 0.726, and 0.666, respectively. In our analysis, the eight-gene signature exhibits a more prominent and reliable prognostic ability, markedly surpassing other established LUAD biomarkers, including TNM stage, TMB, and PD-L1 (CD274) status. As illustrated in Fig. 3g, the multivariate Cox regression analysis yields a HR of 1.48 for the eight-gene signature, accompanied by a p-value of less than 0.001. This statistically robust result strongly suggests that the eight-gene signature is significantly associated with patient prognosis, with each unit increase in its associated expression level or risk score corresponding to a 48% rise in the risk of adverse events. Notably, the pronounced prognostic impact of the eight-gene signature on patient survival remains unaltered even after accounting for the influence of other variables in the multivariate model, demonstrating its significant and independent prognostic value. These findings collectively indicate that the eight-gene signature has a more pronounced and reliable prognostic ability compared to the aforementioned three biomarkers, highlighting the immense potential of our risk model as a novel and valuable tool for clinical prognostic assessment in LUAD.

### Proliferation-cycle gene signatures define risk-related co-expression modules in lung adenocarcinoma

**Fig. 5:**
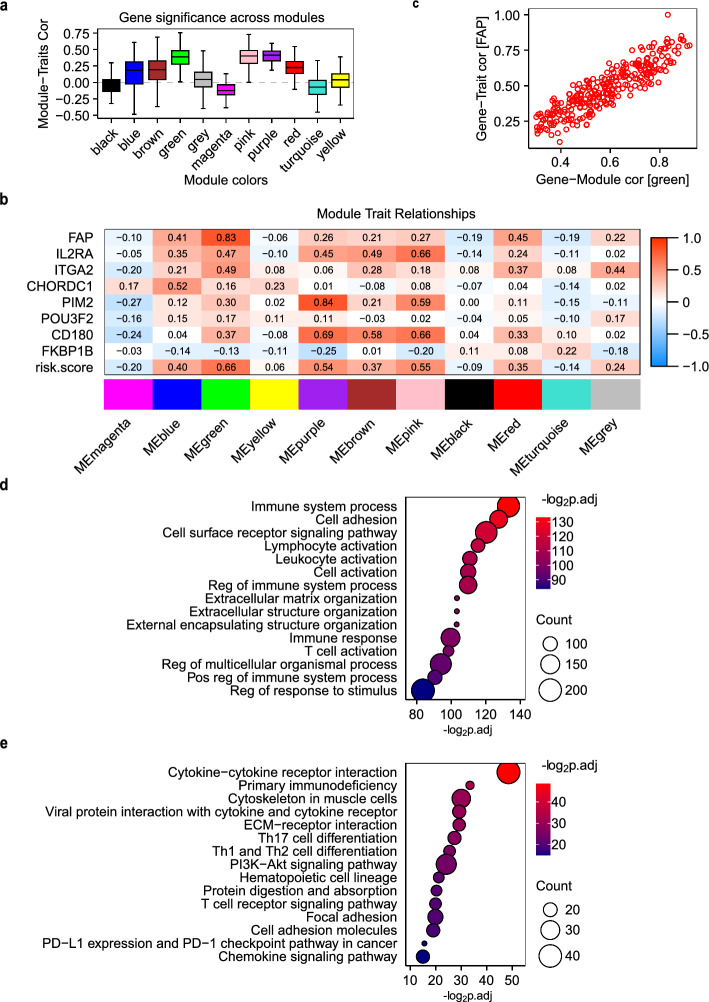
WGCNA analysis correlates Module-Trait associations with risk stratification. **a** Boxplot of Module-Traits correlation across WGCNA modules. **b** Heatmap of module-risk score correlations. **c** Dot plot of Gene-Trait and Gene-Module correlation for *FAP and* the green module. **d**, **e** WGCNA analysis linking FAP expression to immune-related modules. Bubble plot of the top 15 **d** GO and **e** KEGG pathways enriched in genes of the green, purple and pink modules

Leveraging the proliferation-cycle gene signature encompassing eight prognostic risk factors, we implemented Weighted Gene Co-expression Network Analysis (WGCNA) to decipher clinically relevant co-expressed molecular modules impacting tumor prognosis. WGCNA identified 11 gene co-expression modules, with the green, purple, and pink modules exhibiting the strongest associations with the risk model (correlation coefficients: 0.66, 0.54, and 0.55, respectively; Fig. 5a and b). These modules harbored 476 feature genes (Table S2), including *FAP* (green module), *PIM2/CD180* (purple module), and *IL2RA/PIM2/CD180* (pink module) as its module hubs (Fig. 5b). A significant positive correlation was observed between each trait and corresponding module (Fig. 5c), demonstrating robust association. Functional enrichment (green, purple, and pink modules) revealed significant overrepresentation of cell adhesion-related pathways (e.g., focal adhesion, cytokine–cytokine receptor interaction) and immune-regulatory processes (e.g., T cell activation, PD-1/PD-L1 checkpoint pathways) in all three modules (Fig. 5d and e), implicating the involvement of these genes in tumor-immune crosstalk.

**Fig. 6:**
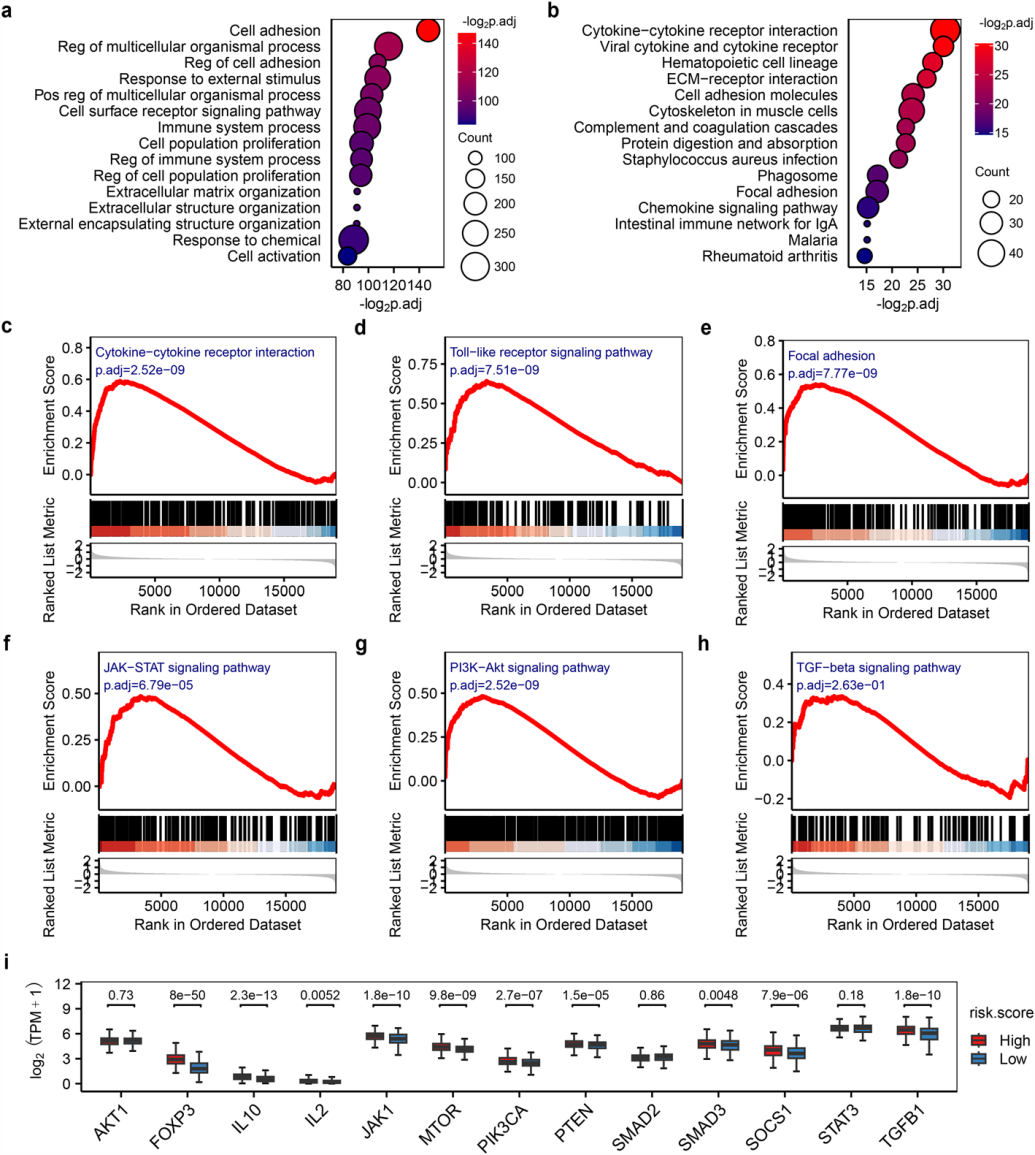
Function and pathway dysregulation associated with risk stratification. **a**, **b** Bubble plot of the top 15 enriched **a** biological processes and **b** KEGG pathways. **c**–**e** GSEA reveals enrichment of genes in **c** Cytokine-cytokine receptor interaction pathway (hsa04060), **d** Toll-like receptor signaling pathway (hsa04620) and **e** Focal adhesion pathway (hsa04510). **f**–**h** GSEA identified significant alterations in three immunosuppressive-related signaling pathways: **f** JAK-STAT (hsa04630), **g** PI3K-AKT (hsa04151), and **h** TGF-β (hsa04350) signaling pathway. **i** Differential expression of immunosuppressive-related genes in JAK-STAT, PI3K-AKT, and TGF-β pathways

Comparative analysis between high- and low-risk groups identified 939 DEGs (546 up-regulated, 393 down-regulated; Table S3). These DEGs, significantly linked to oncological risks, showed marked enrichment in cell adhesion networks (e.g., cell adhesion, focal adhesion, extracellular matrix and structure organization, ECM–receptor interaction), immune and inflammatory relevant pathways (e.g., immune system process, cytokine–cytokine receptor interaction, complement and coagulation cascades, chemokine signaling) (Fig. 6a and b). Notably, enrichment of bacterial infection-related pathways (e.g., *Staphylococcus aureus* infection) suggested that microbiota-driven inflammation could serve as a potential oncogenic cofactor. Concurrently, GSEA corroborated these observations by uncovering significant dysregulation in cytokine–receptor interactions, inflammatory responses, and adhesion pathways within high-risk tumors (Fig. 6c–e).

To gain a more in-depth understanding of the relationship between proliferation-cycle genes and immunosuppression, we conducted a comprehensive examination of the results derived from GSEA. Our analysis revealed that key signaling pathways associated with immunosuppression, namely JAK-STAT (hsa04630), and PI3K-AKT signaling pathway (hsa04151), exhibited significant alterations when comparing the high-risk group with the low-risk group (Fig. 6f, g). Although the TGF-β (hsa04350) signaling pathway had not reached statistical significance, the genes within this pathway were notably enriched in the high-risk group (Fig. 6h). Several key genes in the JAK-STAT (*IL2*, *IL10*, *JAK1*, *SOCS1*), PI3K-AKT (*PIK3CA*, *PTEN*, *MTOR*), TGF-β (*TGFB1*, *SMAD3*, and *FOXP3*) signaling pathways exhibited significant differential expression between high-risk and low-risk groups (Fig. 6i), all of which are critical for immunosuppression. Strikingly, *FOXP3* within the TGF-β signaling pathway exhibited the most pronounced differential expression, suggesting that high-risk LUAD patients may indirectly suppress immune cell activity (e.g., Tregs) via activation of *FOXP3* in this pathway. These findings demonstrate a notable correlation between proliferation-cycle genes and the disruption of immunosuppressive processes, offering valuable insights into the molecular etiopathogenesis involved in the transition from normal cellular conditions to disease-related phenotypes, especially in tumorigenesis and immune-related disorders. At present, the detailed molecular mechanisms underlying this correlation have not been fully determined, and this study marks the beginning of a new era in understanding these mechanisms.

Collectively, these data establish proliferation-cycle genes as critical determinants of the LUAD immune landscape, characterized by dysregulated immune system processes, enhanced inflammatory signaling, and aberrant cell adhesion. These insights enable dual stratification of patients for immunotherapy, offering a precision medicine framework for this malignancy.

### Risk stratification defines distinct TIME profiles

**Fig. 7:**
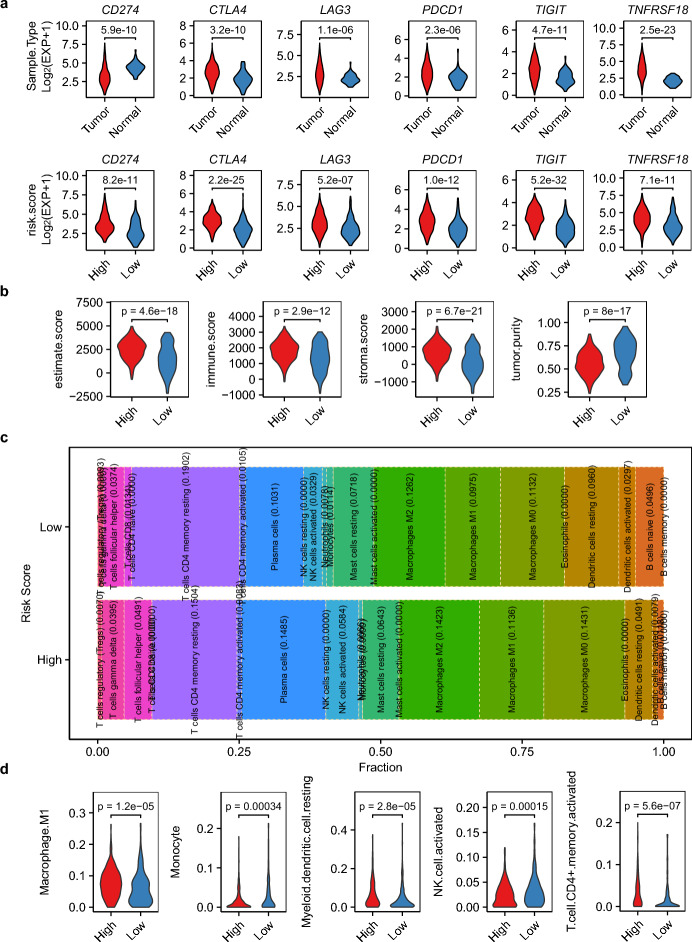
TIME reprogramming by risk signature. **a** Expression of immune checkpoint molecules in risk-stratified patients. **b** ESTIMATE scores quantifying stromal and immune infiltration patterns across risk-stratified patient cohorts. **c** Distribution of 22 immune cell fractions in high- vs. low-risk groups via CIBERSORT analysis. **d** Violin plot showing significant differences (p < 0.001) in five immune cell fractions between risk strata

Proliferation-cycle gene signatures significantly correlate with the TIME, enabling robust stratification of LUAD patients into high- and low-risk groups. Immune checkpoint molecules (*CD274/PDL1, CTLA4/CD152*, *LAG3*, *PDCD1/PD1*, *TIGIT*, *TNFRSF18*) exhibited risk-aligned expression patterns (Fig. 7a), with elevated expression in high-risk patients correlating with impaired T-cell immunity and tumor immune evasion [38, 39]. Notably, risk-based stratification outperformed conventional tumor-normal comparisons (*CD274/PDL1*) in predicting LUAD survival outcomes, highlighting its clinical utility (Fig. 7a).

Further analysis using ESTIMATE revealed pronounced immune cell infiltration in high-risk tumors, as evidenced by higher immune (p = 2.9e−12) and stromal scores, coupled with lower tumor purity (p = 8e−17) (Fig. 7b). CIBERSORT deconvoluted distinct immune cell distributions across risk groups. High-risk tumors showed enrichment of immunosuppressive populations (Tregs) and reduced cytotoxic effectors (CD8+ T cells), whereas low-risk tumors exhibited higher proportions of monocytes (dual phenotype) and anti-tumor CD8+ T cells (Fig. 6c). Compared to patients in the low-risk cohort, those in the high-risk group exhibited diminished CD8+ /Treg cell ratios (2.76 vs. 3.61), elevated M1/M2 macrophage polarization indices (0.48 vs. 0.35), and enhanced activated/resting NK cell distribution patterns (3.26 vs. 2.67). Additionally, when analyzing the average proportions of immune cells across different risk stratification cohorts, we found that the low-risk group exhibited significantly higher fractions of monocytes and activated NK cells compared to their high-risk counterparts (Fig. 7d). These findings suggest that patients in the high-risk group may experience altered immune homeostasis, with potential implications for disease progression and therapeutic responsiveness.

Collectively, proliferation-cycle gene-derived risk stratification provides a mechanistic framework linking tumor proliferation, immune landscape heterogeneity, and therapeutic responsiveness. This model advances precision oncology by enabling tailored immunotherapeutic regimens that are precisely matched to TIME subtypes stratified according to proliferation-associated risk profiles.

### The risk patterns show no obvious linear correlation with TMB

**Fig. 8:**
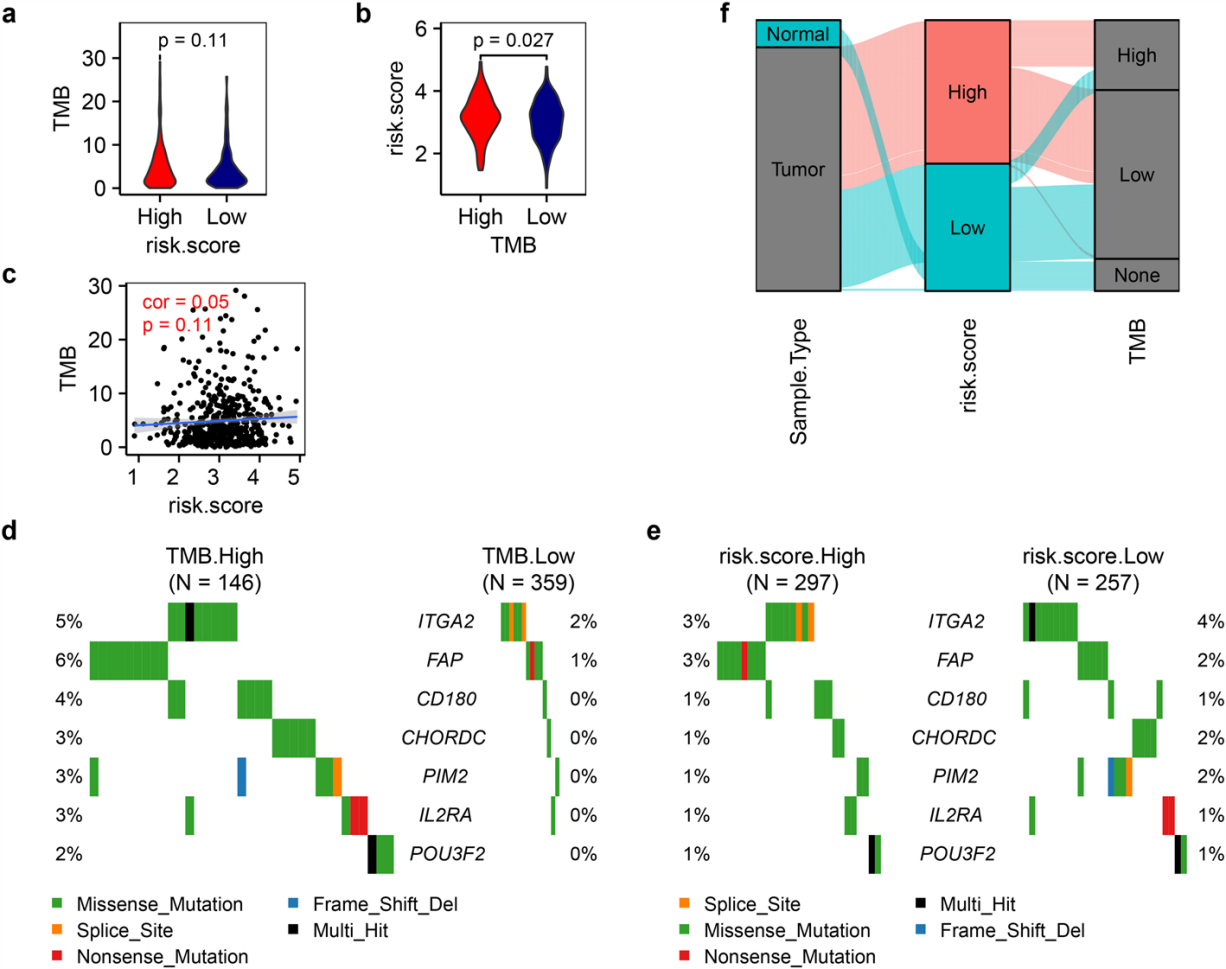
Relationship between TMB and Risk Patterns. **a** Violin plot depicting TMB scores in high and low risk patterns. **b** Violin plot illustrating risk scores in high and low TMB groups. Statistical significance was assessed using a two-tailed t-test for (**a**) and (**b**). **c** Correlation analysis between TMB scores and risk scores. **d**, **e** Tumor somatic mutation landscapes of eight risk factors in LUAD Patients, stratified by **d** TMB score and **e** risk score. **f** Alluvial Diagram representing sample shifts across different sample types, risk score patterns, and TMB score groups

Somatic mutations in tumors can generate aberrant proteins, a subset of which exhibit immunogenicity and are termed neoantigens. These neoantigens, when presented by MHC molecules, can be recognized by T-cell receptors, thereby triggering adaptive anti-tumor immunity. Theoretically, tumors with high TMB harbor a greater likelihood of producing immunogenic driver mutations that enhance immune recognition and response. To interrogate the relationship between risk stratification patterns and TMB, we performed a quantitative analysis of TMB across patient cohorts. Unexpectedly, TMB distribution did not differ significantly between high- and low-risk groups (Fig. 8a). While, high TMB tumors exhibited marginally poorer survival outcomes congruent with elevated risk scores (Figs. 2c and 8b). Risk scores showed only a weak positive correlation with TMB (cor = 0.05, p = 0.11), and this association failed to reach statistical significance (Fig. 8c). In fact, all seven risk-associated genes (no mutation was observed in *FKBP1B*) displayed higher somatic mutation frequencies in the high TMB subgroup (Fig. 8d), while three genes (*ITGA2*, *CHORDC1*, and *PIM2*) surprisingly exhibited slightly elevated mutations in the low-risk cohort (Fig. 8e). These findings highlight that although TMB patterns may offer limited utility for risk stratification, their weak association with risk patterns renders reverse inference unreliable. While our data (Fig. 8c, cor = 0.05, p = 0.11) demonstrate no statistically significant linear correlation between risk patterns and TMB, we recognize that this does not preclude the existence of more complex non-linear relationships. The alluvial diagram (Fig. 8f) suggests potential patterns beyond simple linearity, which warrant further exploration using methods capable of detecting non-linear associations.

Collectively, these observations highlight the limitations of relying solely on TMB as a predictor of immune landscape complexity or therapeutic responsiveness in LUAD. Proliferation-cycle gene signatures encompass additional dimensions of tumor biology beyond mutational burden, warranting integrated multiple approaches in the design of precision immunotherapy.

### FAP and CHORDC1 are promising anti-tumor targets

We have particularly noticed that FAP and CHORDC1 may serve as promising therapeutic targets due to their elevated expression across multiple cancer types. FAP, a cell surface glycoprotein with serine protease activity, plays multifaceted roles in immunosuppression, inflammatory modulation, and tumor progression, as evidenced by its association with poor clinical outcomes [40–43]. Notably, CHORDC1 overexpression significantly correlated with aggressive disease phenotypes and poorer survival across diverse malignancies, validating its potential as a robust prognostic biomarker [44]. These findings collectively suggest a potential rationale for designing FAP- or CHORDC1-targeted therapeutics to improve survival outcomes, though experimental validation is needed to further confirm their clinical utility.

## Discussion

Emerging evidence highlights the critical roles of cell proliferation and cell cycle-related genes in driving tumorigenesis, progression, metastasis, and modulating anti-tumor responses [45, 46]. However, systematic analysis of these genes in LUAD remains lacking, particularly their association with TIME infiltration patterns. Elucidating such relationships is essential for advancing immunotherapy strategies and enhancing clinical outcomes.

Through tumor-normal transcriptomic comparisons, we identified eight cell proliferation/cell cycle-related risk factors (*FAP*, *IL2RA*, *ITGA2*, *CHORDC1*, *PIM2*, *POU3F2*, *CD180,* and *FKBP1B*) in LUAD. Among them, seven genes (*FAP*, *IL2RA*, *ITGA2*, *CHORDC1*, *PIM2*, *POU3F2*, and *CD180*) were significantly overexpressed. These genes correlate with aggressive tumor behavior and poor prognosis across multiple cancers [47–53], making them promising diagnostic biomarkers and therapeutic targets. For instance, FAP—a serine protease overexpressed in solid tumors—has demonstrated preclinical efficacy as a CAR-T cell target [54, 55]. Similarly, CHORDC1’s tumor-specific expression positions it as a candidate for targeted peptide/antibody-based therapies. Despite limited initial therapeutic exploration, IL2RA, ITGA2, PIM2, POU3F2, and CD180 exhibit consistent overexpression in LUAD tumors—coupled with roles in tumor proliferation and TIME modulation—strongly supporting their prioritization as potential candidate druggable targets.

Our risk model integrating the eight proliferation-cycle genes demonstrates robust predictive value for LUAD outcomes (p = 6.2e−05), offering critical insights into tumor biology and clinical management. By stratifying patients based on risk patterns, we revealed distinct immune infiltration patterns linked to clinical outcomes. For example, we found high-risk groups exhibited elevated Tregs cells, while CD8+ T cells and activated NK cells were depleted—a disparity linked to immunosuppressive TIME remodeling (Fig. 7d). This integration of risk stratification and immune profile provides a framework for personalized treatment selection, particularly in identifying patients most likely to benefit from immune inhibitors or targeted therapies. These findings highlight the therapeutic potential of targeting proliferation-cycle associated risk factors to reinvigorate anti-tumor immunity, particularly through CD8+ T cell and NK cell activation, aligning with emerging strategies to overcome immunotherapy resistance.

To explore the potential for translating these insights into actionable therapies, we identified FAP and CHORDC1 as very promising candidate targets. This enables us to design anti-tumor peptide/antibody therapeutics targeting these proteins in the future through ProteinMPNN [56] and AlphaFold3 [57]. This approach integrates risk stratification, immune microenvironment remodeling, and novel targets discovery, fulfilling critical gaps in LUAD immunotherapy research.

While the risk model demonstrates promising predictive performance for LUAD, its clinical implementation faces several challenges. First, the absence of standardized data across institutions—arising from variations in sample collection, processing protocols, and sequencing platforms—may compromise the reproducibility of gene expression profiling. Second, cost-effectiveness remains a critical concern, particularly in resource-limited settings where high-throughput sequencing may not be feasible. Third, translating identified therapeutic targets into clinical practice requires rigorous preclinical and clinical validation to assess efficacy and safety. Addressing these challenges through collaborative standardization initiatives, prospective multicenter validation, and cost-effective assay development will be essential for translating these findings into actionable clinical strategies.

Collectively, our work identifies proliferation-cycle gene signatures as robust biomarkers for risk stratification in LUAD, delineates their association with TIME heterogeneity, and reveals their potential as therapeutic targets. This work provides a scientific framework demonstrating that proliferation-cycle gene signatures orchestrate patient survival outcomes through risk stratification and TIME remodeling. Future studies will prioritize exploring the therapeutic potential of these genes as actionable targets for LUAD.

## Conclusions

In summary, this study revealed that proliferation-cycle gene signatures play pivotal roles in predicting the immune landscape, prognosis, and therapeutic strategies for LUAD. Notably, these cell proliferation and cell cycle-related genes emerged as robust indicators for lung cancer diagnosis and prognosis. Early screening and risk model-based monitoring facilitated the identification of high-risk populations, enabling timely intervention and tailored treatment approaches. Furthermore, our investigation into the impact of these genes on the TIME deepened our understanding of tumorigenesis, paving the way for innovative tumor prevention and treatment strategies.

## Limitations

First, the reliance on RNA-seq data for our risk model poses challenges in resource-constrained clinical environments. Transcriptomic profiling typically requires high-throughput sequencing infrastructure, specialized bioinformatics pipelines, and substantial costs, which may not be feasible in low- or middle-income countries or community hospitals. Second, in-depth functional studies are lacking, hindering understanding of how these genes modulate the TIME and impact LUAD progression, which is crucial for the development of targeted therapies. Third, qPCR validation in cell lines may be biased due to their artificial nature and genetic changes over time; validation in patient samples is absent, so results may not reflect the real tumor environment. Lastly, the study focuses only on proliferation-cycle related genes, potentially missing other key regulators in LUAD. Fourth, the translational potential of these proliferation-cycle gene signatures as actionable therapeutic targets for LUAD requires rigorous validation through multi-modal experimental paradigms, including in vitro functional assays (e.g., CRISPR-mediated gene editing), in vivo preclinical testing (e.g., PDX models), and early-phase clinical trial design to establish a clear causative link between target modulation and patient survival outcomes.

## Supplementary Information

Supplementary file 1: Table S1. DEGs associated with human cell proliferation and cell cycle.

Supplementary file 2: Table S2. Feature genes of the green, purple, and pink modules.

Supplementary file 3: Table S3. DEGs between high and low risk score groups.

## Data Availability

Raw RNA-Seq data analyzed were downloaded from The Cancer Genome Atlas (TCGA, [https://portal.gdc.cancer.gov]). Other data are provided as supplementary information.

## Abbreviations

AUC: Area under curve
CDKs: Cyclin-dependent kinases
CIBERSORT: Cell-type Identification By Estimating Relative Subsets Of RNA Transcripts
DEGs: Differential Expressed Genes
ESTIMATE: Estimation of STromal and Immune cells in MAlignant Tumours using Expression data
GEO: Gene Expression Omnibus
GO: Gene Ontology
GSEA: Gene Set Enrichment Analysis
HRs: Hazard ratios
IHC: Immunohistochemistry
KEGG: Kyoto Encyclopedia of Genes and Genomes
LUAD: Lung adenocarcinoma
LUSC: Lung squamous cell carcinoma
NCBI: National Center for Biotechnology Information
ROC: Receiver operating characteristic
RSEM: RNA-Seq by Expectation Maximization
STAR: Spliced Transcripts Alignment to a Reference
TAMs: Tumor-associated macrophages
TCGA: The Cancer Genome Atlas
TIME: Tumor immune microenvironment
TMB: Tumor mutational burden
TPM: Transcripts Per Million
Tregs: Regulatory T cells
WGCNA: Weighted Gene Co-expression Network Analysis
